# Electrically assisted cycling for individuals with type 2 diabetes mellitus: protocol for a pilot randomized controlled trial

**DOI:** 10.1186/s40814-019-0508-4

**Published:** 2019-11-23

**Authors:** Jessica E. Bourne, Angie Page, Sam Leary, Robert C. Andrews, Clare England, Ashley R. Cooper

**Affiliations:** 1 Centre for Exercise, Nutrition and Health Sciences, School of Policy Studies, University of Bristol, 8 Priory Road, Bristol, BS8 1TZ UK; 2 NIHR Bristol Biomedical Research Centre, University Hospitals Bristol NHS Foundation Trust and University of Bristol, Bristol, UK; 3 Institute of Biomedical and Clinical Sciences, Medical Research, University of Exeter Medical School, RILD Level 3, Barrack Road, Exeter, Devon EX2 5DW UK

**Keywords:** Type 2 diabetes mellitus, Electrically assisted cycling, Intervention, Physical activity

## Abstract

**Background:**

The global incidence of type 2 diabetes mellitus (T2DM) is increasing. Given the many complications associated with T2DM, effective management of the disease is crucial. Physical activity is considered to be a key component of T2DM management. However, people with T2DM are generally less physically active than individuals without T2DM and adherence to physical activity is often poor following completion of lifestyle interventions. As such, developing interventions that foster sustainable physical activity is of high priority. Electrically assisted bicycles (e-bikes) have been highlighted as a potential strategy for promoting physical activity in this population. E-bikes provide electrical assistance to the rider only when pedalling and could overcome commonly reported barriers to regular cycling. This paper describes the protocol of the *PEDAL-2* pilot randomized controlled trial, an e-cycling intervention aimed at increasing physical activity in individuals with T2DM.

**Methods:**

A parallel-group two-arm randomized waitlist-controlled pilot trial will be conducted. Forty individuals with T2DM will be randomly assigned, in a 1:1 allocation ratio, to an e-cycling intervention or waitlist control. Recruitment and screening will close once 20 participants have been randomized to each study arm. The intervention will involve e-bike training with a certified cycle instructor and provision of an e-bike for 12 weeks. Data will be collected at baseline, during the intervention and immediately post-intervention using both quantitative and qualitative methods. In this trial, the primary interests are determination of effective recruitment strategies, recruitment and consent rates, adherence and retention and delivery and receipt of the intervention. The potential impact of the intervention on a range of clinical, physiological and behaviour outcomes will be assessed to examine intervention promise. Data analyses will be descriptive.

**Discussion:**

This paper describes the protocol for the *PEDAL-2* pilot randomized controlled trial. Results from this trial will provide information on trial feasibility and identify the promise of e-cycling as a strategy to positively impact the health and behaviour of individuals with T2DM. If appropriate, this information can be used to design and deliver a fully powered definitive trial.

**Trial registration:**

ISRCTN, ISRCTN67421464. Registered 03/01/2019.

## Background

Type 2 diabetes mellitus (T2DM) is one of the fastest growing global diseases [1]. In the UK, the prevalence of diagnosed T2DM is expected to rise from 3.7 million individuals in 2017 to 5 million by 2025 [2]. T2DM is associated with micro- and macrovascular complications and it is estimated that 10% of the NHS annual UK budget is spent on the treatment of diabetes and its associated complications [3].

Engaging in regular physical activity is a key component of T2DM management [4, 5] that can lead to lowering of glycated haemoglobin (HbA1c) concentration [6]. Physical activity also independently reduces cardiovascular risk factors and contributes to weight loss [7]. However, individuals with T2DM have lower levels of physical activity than individuals without diabetes [8]. Enrolment in structured lifestyle interventions is effective at increasing physical activity; however, when left to self-manage physical activity behaviour post-intervention, individuals often return to an inactive state [9–11] or fail to engage in physical activity of sufficient intensity or volume to positively impact glucose control [12]. With the increasing prevalence of T2DM, there is a need to develop sustainable interventions that can foster independent sustainable physical activity at an intensity that is high enough to generate positive health outcomes.

Active travel represents a potential means through which to increase physical activity. In the UK, approximately 50% of all journeys made by car, both for commuting and leisure purposes, are between 1 and 5 miles in length [13]. Given that individuals report a willingness to actively travel distances of 0.5–2 miles by walking [14] and 1.5–4.7 miles by cycling [15], it is feasible that these short motorized journeys could be replaced by active means and potentially increase physical activity [16]. For example, in healthy adults, active travel, particularly commuting, is associated with an increase in physical activity [17], reduced likelihood of diabetes diagnosis [18], lower body mass index (BMI) [19] and improved cardiovascular health [18]. Among individuals with T2DM, active commuting is associated with increased physical activity and lower BMI [20]. While both walking and cycle commuting serve to increase physical activity, research suggests that cycling may provide greater health benefits than walking [21], potentially due to the higher intensity of activity associated with cycling in comparison to walking [22].

Despite these positive health outcomes, rates of active commuting in the UK, especially cycling, are low in both the general population and among individuals with T2DM [20]. There are a number of barriers to regular cycling that could discourage engagement including physical constraints associated with hilly terrain and poor physical fitness as well as a lack of time and the distance people have to travel to work [23]. These barriers may be accentuated in individuals with T2DM given their overall lower levels of physical activity.

Electrically assisted bicycles (e-bikes), also known as Pedelecs, could help to overcome such barriers to regular cycling by providing electrical assistance only when the rider is pedalling leading to increased speed with reduced physical exertion compared to conventional cycling. This extra assistance is believed to be the main motivator for the increased popularity seen in e-bikes over recent years, particularly among middle- and older-aged adults [24, 25]. The provision of an e-bike has been associated with an increase in self-reported physical activity behaviour of approximately 353 min per week among inactive individuals [26]. Furthermore, evidence suggests that e-cycling can replace the sedentary behaviour of motorized transportation [27]. Despite the increased assistance, research indicates that among physically inactive adults riding an e-bike provides physical activity of at least a moderate-intensity (> 3 METs) and can lead to improvements in cardiorespiratory fitness [28] and glucose disposal rate [25].

Among individuals with T2DM, a feasibility study reported that the provision of an e-bike for 5 months led to a 10% increase in power output, a sign of increased fitness [29] likely to be the result of increased physical activity. Furthermore, e-cycling was perceived as enjoyable with 14 of the 18 participants purchasing the e-bikes at the end of the study. This study highlights the promise of e-cycling as a means of increasing physical activity in individuals living with T2DM. Building on this work, an adequately powered randomized controlled trial (RCT), comparing an e-cycling intervention to a control group is needed to assess the effectiveness of e-cycling on health and behavioural outcomes among individuals with T2DM. However, there is currently insufficient evidence to support a full-scale RCT, nor are there data to allow estimation of appropriate sample size. Therefore, a pilot RCT is needed to determine the feasibility of conducting such a trial and to provide key information needed for the design of a full-scale RCT trial, if warranted.

As such, the *primary* aim of this study is to test the feasibility of conducting a randomized e-cycling intervention among individuals with T2DM. In order to address this aim the primary objectives are to (1) identify effective methods of recruiting individuals with T2DM; (2) determine participants’ willingness to be randomized, study retention rates, adherence to the intervention and data collection methods and harmful outcomes; (3) assess intervention fidelity; (4) qualitatively examine the acceptability of the intervention and study procedures to participants and instructors; and (5) qualitatively examine participants experiences of e-cycling. The *secondary* aim is to examine the association between the intervention and outcome measures to determine intervention promise. Accordingly, the secondary objective is to collect data on a range of individual health and behaviour outcomes in order to estimate the potential effect of the intervention (based on condition allocation) to inform outcome selection in future trials.

## Methods

### Study design

**Fig. 1:**
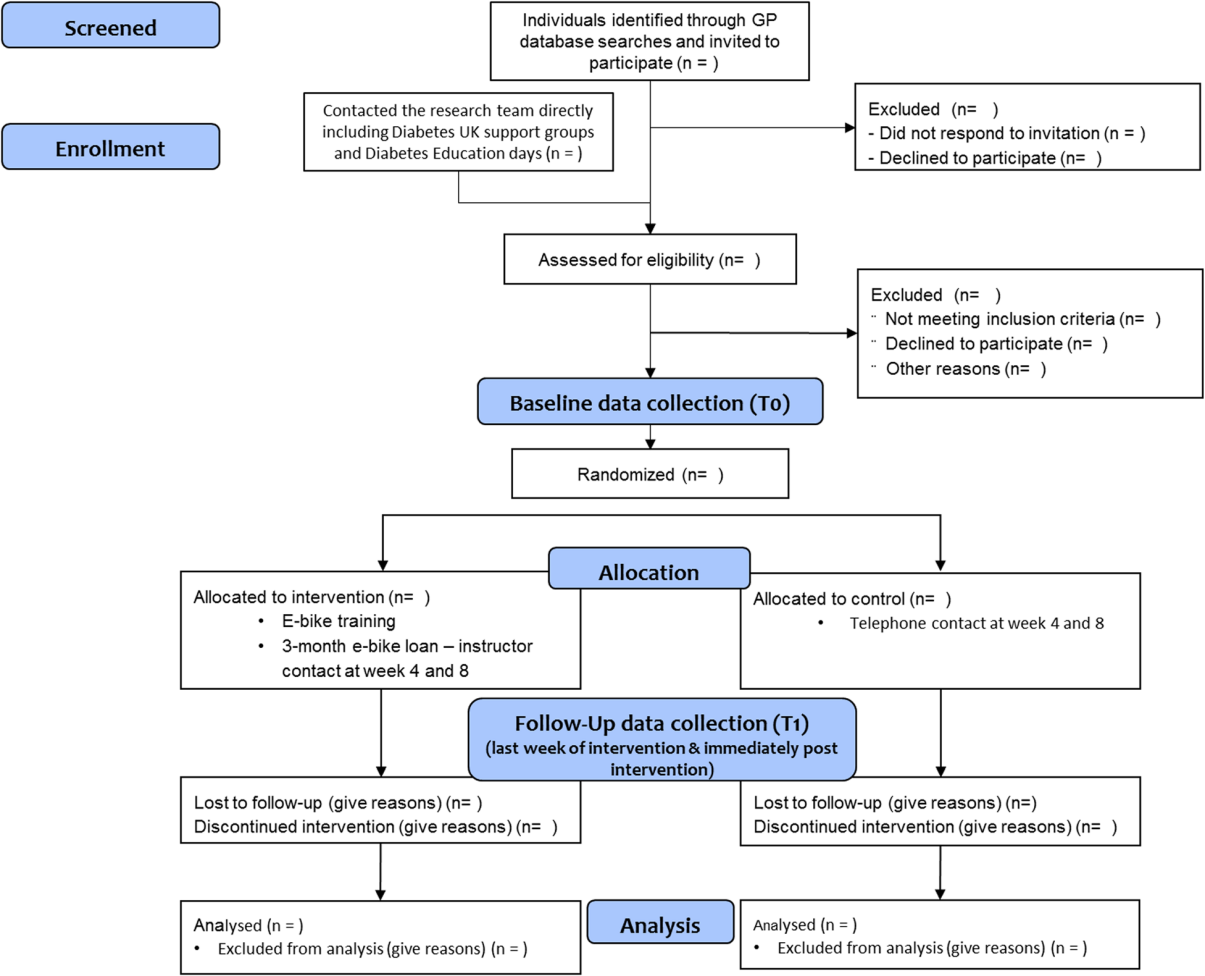
Flow diagram of the PEDAL-2 trial

This pilot study is a parallel-group 2-arm, randomized waitlist-controlled trial comparing an e-cycling intervention (PEDAL-2) against a standard-care waitlist control among individuals with T2DM. A total of 40 participants will be randomized in a 1:1 allocation ratio to the two study arms. The single-centre trial will be conducted in the city of Bristol, England. Recruitment for the trial will begin in March 2019. The majority of measures will be collected at baseline (time 0 (T0)) and immediately following the intervention period (T1). In addition, data will be collected in the final week of the e-cycling intervention (physical activity and travel behaviour) and throughout the intervention (e-cycling time and distance). Figure 1 shows the study flow diagram and Additional file 1 provides the SPIRIT checklist for reporting intervention trials.

### Ethical approval and data protection

The project has been approved by the NHS Health Research Authority South West/Central Bristol Research Ethics Committee (Ref: 18/SW/0164) and is sponsored by the University of Bristol. Any amendments to the protocol will be authorized by the sponsor (University of Bristol) and submitted to the REC and HRA for approval. All data collected in this study will be maintained and stored in strict accordance with the data protection regulations. All patient identifiable information (i.e. names, addresses, dates of birth etc.) will be stored in a database separate from the database that holds anthropometric measures, results of blood tests, physiological measures and travel and physical activity data. Personal data stored on NHS or university computers will be password protected and only the study investigators will have access to the passwords. Personal data on paper files will be stored in a locked filing cabinet within the Biomedical Research Centre at the University of Bristol.

### Participant recruitment

Recruitment will occur over three settings. These recruitment settings include (1) primary care practices, (2) diabetes education days in Bristol run by the Diabetes and Nutrition service and (3) Diabetes UK Support Groups in Bristol. All primary care practices in the Bristol, North Somerset and South Gloucester Clinical Commissioning Group will be invited to act as participant identification sites for the study. All practices that wish to act as participant identification sites will conduct databases searches and send study information to all potentially eligible patients. At diabetes education days, nurses will provide study information sheets to all individuals attending the session. These education days occur approximately once a month in Bristol. At the four Diabetes UK support groups in Bristol, information about the study will be disseminated by a member of the research team. Individuals will also be provided with study information sheets. Individuals who wish to participate in the study will be asked to contact the study team directly by telephone, in writing or by email. Individuals who contact the research team will be asked how they learnt of the study. Eligibility will be determined over the telephone. Individuals deemed eligible will be asked to get clearance to engage in physical activity and have their blood pressure taken by their general practitioner. All participants deemed eligible for the study at this point will be invited for baseline testing. Table 1 outlines the recruitment and assessment schedule for *PEDAL-2*.

**Table 1:**
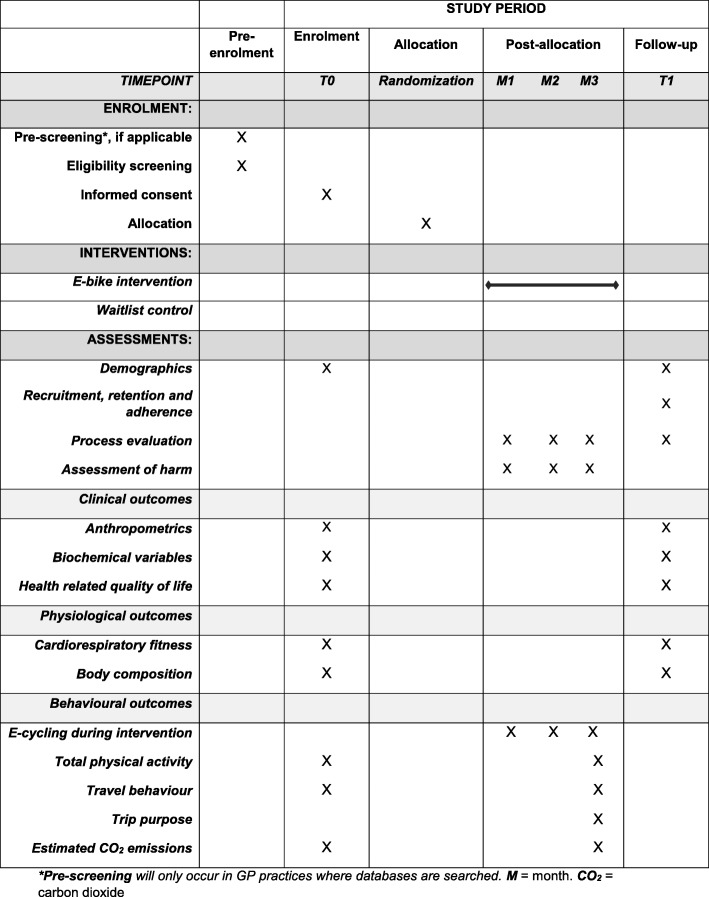
PEDAL-2 SPIRIT diagram displaying study recruitment and measures schedule

*Pre-screening will only occur in GP practices where databases are searched. *M* month, *CO*_*2*_ carbon dioxide

### Eligibility

Individuals will be eligible to participate in the trial if they meet the following inclusion criteria:

- Clinical diagnosis of type 2 diabetes mellitus
- Aged 30–70 years

Individuals will be ineligible to participate if they meet any of the following criteria:

- Currently engage in ≥ 150 min of moderate to vigorous physical activity per week (assessed by the Get Active Questionnaire [30])
- Currently taking exogenous insulin
- Have uncontrolled hypertension (systolic blood pressure > 160 mmHg and/or diastolic blood pressure > 90 mmHg), for which they are not taking medication
- Have had a myocardial infarction or stroke within the past 6 months or have evidence of end-stage renal failure or liver disease
- Have any other contra-indications to exercise
- Are not cleared to engage in physical activity by their general practitioner
- Are unable to read and communicate in English

### Sample size

We aim to recruit and randomize 40 individuals for the pilot study. This sample size is based on recommendations for pilot studies which aim to provide an estimation of a standard deviation for use in the sample size calculation to inform a larger randomized controlled trial [31, 32]. There are no explicit targets regarding the number of individuals to be recruited or screened as we are investigating de novo the feasibility of recruitment from primary care. Based on recruitment rates in a similar population and region, we would anticipate a recruitment rate of approximately 30% [33]. Recruitment rates are anticipated to be slightly lower for a cycling intervention compared to a combined diet and exercise intervention, with a previous e-cycling feasibility study reporting a recruitment rate of approximately 20% [29]. Recruitment and screening will close when 20 participants have been randomized to each of the two study arms. The number of individuals invited to participate in the study and the numbers recruited will be recorded. Based on a previous feasibility study, a retention rate of approximately 80% is anticipated [29].

### Consent

Once participants have been identified as eligible to participate in the study, they will be booked in for their baseline data collection visit at the University of Bristol (T0). At this first face to face contact a member of the research team will outline the study procedures, as per the information sheet. Participants will be advised that the study is voluntary and that they have the right to withdraw at any time, without the need for explanation. After this, individuals who wish to participate will be asked to read, complete and sign a consent form, which will be countersigned by the member of the research team obtaining consent.

### Allocation and randomization

Randomization will occur after consent is obtained and baseline (T0) data has been collected. Forty individuals will be stratified based on sex and then randomly assigned to either the e-cycling intervention or waitlist control in a 1:1 allocation ratio. Permuted blocks of random size will be used. The Biomedical Research Centre data manager will generate the random allocation sequences which will be accessible through a password protected web page. Researchers will enter the participant ID code and sex into the web page, and a random allocation will be issued. Researchers will be aware of the group allocation. Participants will be informed of the group allocation via telephone by a member of the research team. Blinding of intervention allocation will not be possible for any participant involved in the trial. A maximum of 20 participants will be randomized to each of the trial arms.

### PEDAL-2 intervention

#### Intervention content

Intervention content was designed using qualitative data from one-to-one interviews with participants who took part in an e-cycling feasibility study conducted in the summer of 2016 [29]. Interviews were used to identify barriers and enablers to e-bike use which were then categorized using the Theoretical Domains Framework [34]. These barriers and enablers were mapped onto intervention functions [35], and behaviour change techniques (BCTs; i.e., the active ingredients of an intervention) deemed most appropriate to deliver the intervention functions were identified. In addition, behaviour change techniques identified in the literature as significantly impacting upon general physical activity behaviour in individuals with T2DM were incorporated into the intervention design [36, 37]. The utility of these BCTs in the current intervention was considered with regard to affordability, practicality, effectiveness, cost-effectiveness, acceptability, side-effects/safety, and equity (the APEASE criteria [35]). In the present study, 17 behaviour change techniques will be incorporated into the intervention (see Additional file 2 for intervention content and associated behaviour change techniques incorporated into PEDAL-2).

#### Instructor training

Four instructors from Life Cycle UK, a Bristol-based cycling charity who specialize in bicycle training, will deliver the intervention. All instructors will be disclosure and barring service checked and first aid qualified. Instructors are fully qualified National Standard cycle instructors, and so instructor training will focus on the behavioural aspects of the intervention content. In training session one (3 h), instructors will be taught how to communicate with participants in a way to promote and encourage behaviour change. Training session two (2 h) will be focused on reviewing the intervention manual and discussing the importance of completing intervention activities specified in the manual. Instructors will be provided with checklists to record and monitor intervention activities and report changes to the intervention content.

#### E-bike training

Following baseline measures (T0) participants allocated to the intervention will complete e-bike training at Life Cycle UK. The training will consist of two one-to-one sessions. Training session one is mandatory and will follow the National Standard for Cycle Training guidelines for level 1 and 2. Example activities include demonstration of safety equipment, starting, stopping, making U-turns, and demonstrating decision-making and safe riding strategy. Individuals’ previous cycling experience will be considered when conducting the cycling-specific training.

Training session 2 will be optional and will occur within 2 weeks of session one. The instructor and participant will discuss the need or desire for session 2. Session 2 will provide participants with an opportunity to practice e-cycling skills with the instructor. Busier roads and complex junctions will be incorporated into the session if desired by the participant. Training sessions 1 and 2 will last approximately 2 hours each. Throughout the sessions, the instructors will provide participants with feedback on their e-cycling and give verbal encouragement. Practical e-cycle training will be followed by a discussion in which instructors will help participants identify cycle routes, encourage participants to think about where and when they plan to ride the e-bike and to set specific e-cycling goals. Participants will be encouraged to monitor their e-cycling and will be provided with a log-book to track activity. Alternatively, instructors will assist the participant in setting up a mobile tracking application (Garmin Connect mobile). Instructors will encourage participants to think about potential barriers to e-cycling that could arise and brainstorm strategies to overcome these barriers. Instructors will also discuss the potential health, social and environmental consequences of e-cycling. Participants will be invited to join a private social media group to share their experiences and ride ideas with other individuals participating in the intervention. Instructors will coordinate this group.

Following the training (1 or 2 sessions depending on participants demonstration of appropriate skill level and confidence) participants will be provided with an e-bike to take home. E-bikes can be ridden home or, if desired, transportation of the e-bike will be provided by Life Cycle UK. Upon taking the e-bike home, participants will be provided with the following:

- Maps of cycle routes in the area
- Instructions of a call out maintenance service in case of breakdown
- Helmet, pannier, bike lock and lights
- Garmin edge 130 GPS device to use and track cycling activity.

#### E-bike loan

Participants will be loaned an e-bike for 12-weeks. Participants will be informed that the e-bike is for use by themselves and not to be lent to friends or family. During this time, participants will be instructed to use the e-bike as they desire, this means that no specific daily or weekly cycling frequency or distance targets will be imposed on participants by the researchers. Four weeks after taking the bike home, participants will attend a ‘refresher’ session with their instructor (session 3). This session will take place at a location of the participant’s choice (i.e., at their home or in the local community) and will last approximately 2 h. The content of the session will depend on the participant’s needs but will include practicing riding skills on established or new routes and a review of participants e-cycling activity as well as action planning and goal setting for future rides. At week 8, the instructor will contact the participant by telephone to discuss the participants progress, barriers to e-cycling that have arisen, and strategies used to overcome them and e-cycling goals for the upcoming month (session 4). At the end of week 12, participants will be asked to return the e-bike to Life Cycle UK headquarters or an instructor will collect the e-bike from the participant’s preferred location. Throughout the loan period, Life Cycle UK will provide a call out e-bike maintenance service. If required, participants are instructed to call the maintenance number and a Life Cycle UK mechanic will come and repair the e-bike.

#### Control group

Individuals randomly assigned to the waitlist control after baseline data collection (T0) will receive two phone calls from the researcher at approximately week 4 and 8 in order to maintain engagement in the study. During these phone calls, the researcher will direct participants to diabetes support groups and additional diabetes services being offered in the local community, in line with standard-care procedures. After post-intervention data collection (T1) these individuals will be offered training session 1 and loaned an e-bike for 3 months. Sessions 2, 3 and 4 will not be conducted. Participants will be asked to report any contact they have with other individuals in the study to ensure no contamination between conditions has occurred.

### Measures

#### Feasibility and acceptability

The following information will be recorded to assess the feasibility of recruitment through GP practices: the number of GP practices approached, the number of practices that agree to participate as participant identification sites, the number of individuals identified through database searches and the response rates. In addition, information on the number of individuals that attend diabetes education days and the Bristol Diabetes UK support group will be recorded. Recruitment rates from the three recruitment settings, consent rates and willingness to be randomized will also be recorded. Retention rates will be determined based on the number of individuals that complete the intervention and follow-up measures. Adherence rates to study procedures will be recorded. The acceptability of the intervention and data collection methodology will be explored through semi-structured one-to-one interviews with instructors and all study participants. These interviews will be conducted by a member of the research team. Interview questions for instructors will focus on factors that impact intervention delivery, including intervention content, facilities, time and burden. Interview questions for participants will focus on thoughts and feelings regarding participation in the intervention and data collection processes. The project team will track the costs and resources required in preparation for running the intervention. Life Cycle UK will track the staff costing of intervention delivery and from the maintenance service.

#### Process evaluation

We will evaluate whether the intervention was delivered and received as intended (implementation). This will be achieved through completion of intervention checklists by Life Cycle UK instructors and through semi-structured interviews with instructors and participants in the intervention group at the end of the intervention. Intervention intensity, recorded by instructors, will be determined through recording of the number of intervention sessions attended by participants as well as the volume of email and telephone contact between instructors and participants. To explore the mechanisms of impact, semi-structured interviews will be conducted with participants in the intervention group to identify barriers and enablers to engaging in e-cycling. These interviews will help to identify how the intervention impacts behaviour and to determine contextual factors that may influence the intervention.

#### Assessment of harm

Participants will be asked to report adverse events resulting from e-cycling (e.g., musculoskeletal problems, falls or road traffic accidents) by calling the study phone line. The number and types of adverse events will be reported. Adverse events that mean the participant is unable to continue with the intervention will also be documented under retention rates. Qualitative interviews will be used to explore any unintended consequences that arise from participation in the study.

#### Outcome measures

Clinical outcomes are those deemed to be of importance to clinicians in the treatment of T2DM. These outcomes will be assessed at baseline (T0) and immediately post-intervention (T1) and include the following:

*Anthropometrics*. Body weight will be assessed to the nearest 0.1 kg using digital scales (TANITA Corp, Tokyo, Japan) and height will be assessed to the nearest 0.1 cm (SECA, 700 SECA, Hamburg, Germany). These measures will be used to calculate BMI (kg/m^2^). Waist circumference will be measured using a non-stretch tape measure to the nearest 0.1 cm, based on World Health Organization guidelines [38].

*Biochemical variables*. Baseline blood samples will be obtained by cannulation of the antecubital fossa from individuals in a fasted state (≥ 8 h overnight fast) to measure glucose, insulin, glycated haemoglobin (HbA1c), lipids (total cholesterol, high-density lipoprotein cholesterol, low-density lipoprotein cholesterol and triglycerides) and C-reactive protein. A total of 8 mL of blood will be taken at this time. After baseline blood samples participants will complete an oral glucose tolerance test (OGTT) which will involve consuming 113 mL of Polycal (Nutricia Advanced Medical Nutrition, Trowbridge, UK) and 87 mL of water, equivalent to 75 g of anhydrous glucose, within 5-min. Further 7 mL blood samples will be drawn at 15-, 30-, 45-, 60-, 90-, and 120-min intervals. The first 5 mL of each draw will be discarded and 2 mL of blood taken for the analysis of glucose and insulin. The intravenous cannula will be kept patent through flushing with 5 mL 0.9% NaCI (B. Braun, Sheffield, UK). All blood samples will be transported immediately to the Bristol Royal Infirmary commercial laboratory and stored at − 80 °C until analysed. Samples will be analysed individually as soon as possible after delivery. Glucose, insulin, lipids and C-reactive protein will be analysed using a Roche Cobas C701 analyzer (Roche Diagnostics, Rotkreuz, Switzerland) and HbA1c will analysed using affinity chromatography. Basal insulin and glucose values will be used to calculate insulin resistance and beta-cell function using the Homeostasis Model Assessment calculator (University of Oxford, Diabetes, Trial Unit). Using values from the OGTT, incremental area under the curve (iAUC) for insulin and glucose will be calculated using the trapezoid rule. Glucose and insulin concentrations during the OGTT will be used to estimate insulin sensitivity using the Matsuda index [39]. The insulinogenic index and oral glucose disposition index will be used to assess beta-cell function. Once samples have been analysed, the remainder of the aliquot will be destroyed by the commercial testing laboratory. Once the study is complete, any remaining samples will be disposed of in accordance with the Human Tissue Authority’s Code of Practice.

*Health-related quality of life*. The Short Form 36 Health Survey (SF-36 [40]) is a 36-item inventory designed to assess health-related quality of life (HRQL) from which 2 measures are derived, a physical component summary and a mental component summary. The physical component summary represents the average of the scales: physical functioning, role limitations due to physical health, bodily pain, and general health subscales. The mental component summary score is the average of the scales which assess energy/fatigue, social functioning, role limitations due to emotional health, and emotional wellbeing subscales. Summary scores are reported in a range from 0 to 100, with a lower score indicating lower quality of life.

Physiological outcomes will be assessed at baseline (T0) and immediately post-intervention (T1) and include the following:

*Cardiorespiratory fitness* will be determined by measuring maximum oxygen uptake (VO_2max_) using a continuous incremental ramp maximal exercise test on an electronically braked cycle ergometer (Lode Excalibur, The Netherlands). The test will start with a 4-min warm-up at 30 W, with participants cycling at a cadence of approximately 60 revolutions per minute (rpm). Following the warm up, the resistance will increase by 1 W every 4 s (15 W per minute). The test will be terminated upon volitional exhaustion or when cadence falls below 50 rpm. Expired gas will be collected continuously by a metabolic cart (Parvomedics TrueOne 2400, Salt Lake City, UT, USA). VO_2max_ is defined as the highest 15-s moving average for VO_2_ (in absolute [l/min] and relative [ml/kg/min] terms). Criteria for achieving VO_2max_ will be (1) respiratory exchange ratio > 1.15, (2) plateau in VO_2_, (3) reaching age-predicted HR_peak_ (220-age); and/or (4) volitional exhaustion. Heart rate will be monitored using a Polar chest strap, which is integrated with the metabolic cart and cycle ergometer software (Lode Exercise Manager). HR_peak_ and peak power output (*W*_peak_) will be recorded as the highest values attained in the test. Twenty minutes after completing the incremental VO_2max_ assessment, participants will complete a supramaximal test to confirm the findings of the incremental assessment. This assessment will follow guidelines outlined by Schaun [41]. The multistage test will consist of a 2-min warm-up at 30 W followed by 1 min at 60% of the incremental VO_2max_ then 110% of incremental VO_2max_ until volitional exhaustion or when cadence falls below 50 rpm [42]. The criteria for achieving VO_2max_ are the same as those reported above. Differences of ≤3% will be considered to demonstrate validation of the incremental VO_2max_ result. The higher of these two values will be taken as VO_2max._

*Body composition*. Dual-energy x-ray absorptiometry (Discovery-A; Hologic, Bedford, UK) scans will be used to assess whole-body and regional fat and lean mass using the manufacturers software. Peripheral quantitative computer tomography (pQCT; XCT 3000 scanner; Stratec, Medizintechnik GmbH, Pforzheim, Germany) will be used to assess intermuscular adipose tissue, muscle density and muscle cross-sectional area (MCSA) of the femur at 33% of the limb length. Data from the pQCT will be analysed using BoneJ [43], a freely available plugin for the software ImageJ2 [44]. Calibrations of these machines will be performed daily following manufacturers guidelines.

#### Behavioural outcomes

*Physical activity* will be measured at baseline (T0) and in the final week of the e-bike intervention. Participants physical activity will be assessed for 7 continuous days using an Actigraph accelerometer (GT3X, Actigraph, Florida, USA). The Actigraph accelerometer will be worn on an elasticated belt around the waist and taken off when sleeping, bathing or swimming. The accelerometer will record raw acceleration data at a sampling frequency of 30 Hz. Raw acceleration data will be processed using Actilife 6 software to reintegrate the data to 10-s epochs. Kinesoft software will be used to generate outcome variables describing physical activity intensity using equations developed by Freedson and colleagues [45], and the frequency and duration of physical activity. In the current study, the Actigraph accelerometer will be used to estimate total time spent in moderate-to-vigorous physical activity (MVPA) before and while having access to an e-bike. This measure has been extensively validated in both laboratory and free-living conditions [46] and has been reported to have a high completion rate in observational studies [47].

*Travel behaviour* will be measured at baseline (T0) and in the final week of the intervention, at the same time as physical activity monitoring. Spatial location will be recorded every 5 s using a personal GPS receiver (QStarz International Co. Ltd., Taiwan). Participants will be asked to wear the GPS receiver during waking hours and recharge the device at night. The device can be worn on the waist or in a pocket as desired. GPS data, in combination with accelerometer data, will be used to estimate (a) the modes of transport used by participants and (b) the amount of time spent in MVPA attributable to e-cycling and other modes of active transport.

Raw GPS data will be downloaded using Qtravel software (Qstarz International Co. Ltd. Taiwan) and extracted as csv files. Raw Actigraph acceleration data will be extracted as csv files using ActiLife 6 software (Actigraph, FL, USA). Data from these devices will be merged by timestamp using an open-source tool, which will (1) classify different modes of transportation and (2) determine the amount of MVPA attributable to different active transport modes in the merged data [48]. This tool has been found to accurately identify active travel 94.6% of the time in a cross-validation study. However, the tool has not been validated with e-cycling. As such, participants will be asked to wear a combined movement sensor and heart rate monitor (Actiheart®, CamNtech, Cambridge, UK) for the same time period as wearing the Actigraph monitor and GPS device. The Actiheart is a waterproof device worn on the left side of the chest and is attached with standard ECG electrodes. Accelerometer and heart rate data will be recorded at 15-s epochs (the shortest epoch available). Fifteen-second acceleration and heart rate data from the Actiheart device will be downloaded using Actiheart 4 software and merged with the GPS data. These data will be imported into ArcGIS for visual inspection and heart rate data will be used to confirm (or otherwise) identification of e-cycling. Once e-bike journeys have been identified, this will enable the estimation of physical activity associated with e-cycling.

#### Trip purpose

In addition to wearing a personal GPS, participants will be asked to complete a 7-day travel diary for the same time period. The travel diary will be adapted from Neves and Brand [49]. Specifically, participants will be asked to record the purpose of the trip, travel mode, the start and end time and the start and end location of each trip. Participants will be asked to classify their trip under one of eight categories: commuting, business, education, escort, shopping, visiting friends, entertainment, and recreation. For each journey, participants will be asked to report the travel mode, walking, cycling, e-biking, bus, train, car (as driver) and car (as passenger). This diary will be used to identify the purpose of trips being made by different transport modes and specifically the purpose of e-bike use.

#### Estimated CO_2_ emissions

Transport-derived CO_2_ emissions will be calculated by multiplying the distance travelled by each motorized mode (determined through GPS and accelerometer data) by the mode’s average emissions factors following the procedure outlined by Neves and Brand [49]. For travel by bus, train and other non-car modes, the total distance travelled in past week, based on GPS and travel diary data, will be multiplied by the average emissions factors based on UK Green House Gases reporting guidelines [50]. For cars, CO_2_ emissions will be estimated by considering the car size (based on engine size), fuel type, vehicle age, number of cold starts (calculated as the number of reported trips) and average speed (using GPS data). These factors underlie the National Atmospheric Emissions Inventory and will be obtained from DEFRA [50].

#### E-cycling during the intervention

The number of e-cycling journeys, the distance travelled on the e-bike and the pattern of e-bike use throughout the 12-week intervention will be determined through use of a cycling GPS unit (Garmin Edge 130). The GPS device will attach directly to the bicycle. Data will be automatically uploaded to the Garmin Connect website via Bluetooth connection with the participant’s phone or manually by the instructor at monthly intervals during meeting times (if the participant does not wish to track their e-cycling via the Garmin Connect website). Participants will be provided with instructions on how to use the device and a power cable to charge the device. The e-bike odometer, which is permanently attached to the bike, will provide a total measure of total distance travelled over the three-months.

### Analysis plan

#### Quantitative analysis

The primary outcomes of this pilot trial include recruitment and consent rates, retention and adherence to study procedures and data provision. Analysis of these data will be descriptive, expressed as frequencies and percentages. Any adverse events will be described appropriately. Characteristics of the sample will be summarized using descriptive statistics (means and standard deviations, medians and interquartile ranges, or frequencies and percentages as appropriate). Descriptive comparisons of these data will be made between the intervention and the waitlist control. Evidence of promise of the intervention (i.e., whether the intervention can lead to changes in outcomes measures) will be examined using comparison of change scores between conditions for all outcome measures (except e-cycling during the intervention). See Table 2 for a description of the outcome measures and proposed analysis plan for each outcome. Effect estimates will be presented with 95% confidence intervals reported; *p* values will not be considered as the study is not powered to detect effectiveness.

**Table 2:** List of PEDAL-2 study objectives, associated outcomes, data collection tools, time point measurements and analysis plan

Study objectives	Outcome	Data collection method/tool	Time point of measurement			Analysis plan
			Baseline (T0)	During intervention	Follow-up (T1)	
1. Identify effective methods of recruiting individuals with T2DM	• # GP practices approached; # that agree to act as PIC • # individuals identified through GP database searches; response rate to information letters • # participants recruited from each recruitment setting • # individuals that consent to be part of the study	Study records	X			Frequencies and percentages
2. Determine participants willingness to be randomized, study retention rates, adherence to the intervention and data collection methods and report harmful outcomes	• # participants retained in study following randomization • # Individuals that complete follow-up testing • # of participants that attend each of the intervention sessions and data collection sessions • # of harmful events	Study records		X		Frequencies and percentages
3. Assess intervention fidelity	• # of training sessions attended by participants and additional contact with instructors • Extent to which intervention content is completed as planned	Intervention checklists		X		Frequencies and Percentages
4. Estimate the potential effect of the intervention on a range of health and behaviour outcomes to inform outcome selection in future trials	• Weight, height, BMI	Tanita digital scales, SECA 700	X		X	Comparison of change scores between conditions
	• Waist circumference	Non-stretch tape measure	X		X	
	• Fasting glucose, insulin, lipids, C-reactive protein, HOMA-IR, HOMA-B	8-mL blood sample	X		X	
	• OGTT outcomes: iAUC for glucose and insulin, Matsuda index, insulinogenic index and oral glucose disposition index	2 mL blood samples at 15, 30, 45, 60, 90, 120 min post 75 g of anhydrous glucose	X		X	Reporting of effect estimates with 95% CI
	• Health-related quality of life: physical and mental summary	Short Form 36 Health Survey [39]	X		X	
	• Cardiorespiratory fitness	Maximum oxygen uptake using cycle ergometer	X		X	
	• Body composition: whole-body fat mass, regional fat mass, whole-body lean mass, regional fat mass	Dual-energy x-ray absorptiometry	X		X	Comparison of change scores between conditions
	• Femur intermuscular adipose tissue, muscle density and muscle cross-sectional area	Peripheral quantitative computer tomography				
	• Total physical activity (time spent in moderate-to-vigorous physical activity)	Actigraph (GT3X)	X		X	Reporting of effect estimates with 95% CI
	• Moderate to vigorous physical due to e-cycling and other modes of active travel	Actigraph (GT3X), Actiheart and QStarz GPS				
	• Transportation modes (walking, cycling, e-cycling, car, bus, train)	Actigraph (GT3X), Actiheart and QStarz GPS	X		X	
	• Trip purpose (e.g., commuting, business, education, escorting, shopping, visiting friends, entertainment, recreation)	Travel diary	X		X	
	• Estimated CO_2_ emissions	Actigraph (GT3X) and QStarz GPS, travel diary following procedures by Neves and Brand [48]	X		X	
	• E-cycling behaviour: # journeys, distance travelled, pattern of e-bike use	Bike odometer and Garmin 130 GPS		X		Mean and SD
Qualitatively examine the acceptability of the intervention and study procedures to participants and instructors	• Acceptability of intervention to participants • Acceptability of study procedures to participants • Acceptability of intervention delivery to instructors	Semi-structured interviews			X	Thematic analysis based on objective
Qualitatively examine participants experiences of e-cycling	• Participants barriers and facilitators to e-cycling	Semi-structured interviews			X	Thematic analysis based on objective

*T2DM* type 2 diabetes mellitus, *GP* general practitioner, *PIC* participant identification center, *HOMA-IR* Homeostasis Model Assessment for assessing insulin resistance, *HOMA-B* homeostatic model assessment for assessing β-cell function, *OGTT* oral glucose tolerance test, *iAUC* incremental area under the curve, *CO*_*2*_ carbon dioxide, *CI* confidence interval, *SD* standard deviation

#### Qualitative analysis

Recordings of interviews will be transcribed verbatim. An abductive approach to data analysis will be taken given the interaction between the data and the study objectives [51], this involves incorporating both deductive and inductive reasoning when analysing the data. Specifically, using the interview guide as a framework, deductive content-based analysis will be conducted to organize initial coding categories based on the study objectives, that is to determine the acceptability of the intervention and study procedures to participants and instructors as well as identify participants barriers and facilitators to e-cycling. Thematic analysis will then be carried out to inductively explore recurring patterns within subcategories. Content will be further delineated into sub themes with similar content. Each transcript will be analysed independently by two researchers. Once complete, the two researchers will compare and discuss coding and categorization. Any disagreements will be discussed and resolved through consensus.

The following progression criteria will be used to guide the decision as to whether to proceed to a definitive trial:

1. At least 20% of potentially eligible individuals express an interest in being part of the study. This criterion is based on previous feasibility work conducted in a similar population [29]. The proportion of individuals that express an interest in the study from each recruitment strategy will be calculated in order to identify the most effective recruitment method and to determine, where appropriate, the number of GP practices, Diabetes Education sessions or Diabetes Support groups that need to be approached to successfully recruit for a future trial. The three methods of recruitment will be compared.
2. At least 80% of eligible individuals (identified through telephone screening and GP study clearance) are successfully randomized
3. Attrition of the pilot trial is low, with a study retention rate of ≥ 80%. This criterion is based on findings from a previous feasibility study conducted in a similar population [29].
4. At least 70% of participants in the intervention group attend at least 60% of the intervention sessions. This criterion is based on previous physical activity interventions conducted in individuals with type 2 diabetes [52].
5. Process evaluation findings suggest that > 80% of participants report the study methodology to be comprehensible and acceptable.

## Discussion

Physical activity is a key component of managing T2DM. However, this population is less physically active than individuals without diabetes. E-cycling has been found to be an acceptable activity in individuals with T2DM; however, more research is needed to examine the feasibility of conducting a randomized controlled trial and to determine if e-cycling demonstrates a potential to positively impact both health and behavioural outcomes. This paper describes the protocol of *PEDAL-2*, a pilot randomized waitlist-controlled trial designed to evaluate the feasibility of conducting an e-cycling intervention in individuals with T2DM. The e-cycling intervention has been developed using previous literature and semi-structured interviews with the target population. It is important to acknowledge potential limitations in the proposed methodology. Specifically, the lack of blinding may create challenges with study retention particularly in the control group, potentially creating bias. This is common to many exercise studies and we have addressed this by offering all control participants the e-bike intervention at the end of the trial period. In addition, this single-centre pilot trial limits the ability to generalize to other cities across the UK or rural areas in which the feasibility and associated outcomes could be different.

Despite these limitations, the data collected in this trial could be used to inform the development of future e-cycling interventions and identify appropriate outcome measures for examination in a definitive trial if deemed appropriate.

## Supplementary information

**Additional file 1.** SPIRIT checklist.

**Additional file 2.** Behavioural Intervention.

## Data Availability

Not applicable.

## Abbreviations

BCT: Behaviour change technique
BMI: Body mass index
CO_2_: Carbon dioxide
HbA1c: Glycated haemoglobin
METs: Metabolic equivalents
MVPA: Moderate to vigorous physical activity
OGTT: Oral glucose tolerance test
pQCT: Peripheral quantitative computer tomography
RCT: Randomized controlled trial
T2DM: Type 2 diabetes mellitus

## References

[1] Ogurtsova K, da Rocha Fernandes JD, Huang Y, Linnenkamp U, Guariguata L, Cho NH (2017). IDF Diabetes Atlas: global estimates for the prevalence of diabetes for 2015 and 2040. Diabetes Res Clin Pract.

[2] Diabetes UK (2017). Diabetes Prevalence 2017.

[3] Hex N, Bartlett C, Wright D, Taylor M, Varley D (2012). Estimating the current and future costs of type 1 and type 2 diabetes in the UK, including direct health costs and indirect societal and productivity costs. Diabet Med.

[4] Inzucchi SE, Bergenstal RM, Buse JB, Diamant M, Ferrannini E, Nauck M (2012). Management of hyperglycemia in type 2 diabetes: a patient-centered approach. Diabetes Care.

[5] Colberg SR, Sigal RJ, Yardley JE, Riddell MC, Dunstan DW, Dempsey PC (2016). Physical activity/exercise and diabetes: a position statement of the American Diabetes Association. Diabetes Care.

[6] Grace A, Chan E, Giallauria F, Graham PL, Smart NA (2017). Clinical outcomes and glycaemic responses to different aerobic exercise training intensities in type II diabetes: a systematic review and meta-analysis. Cardiovasc Diabetol.

[7] Lin X, Zhang X, Guo J, Roberts CK, McKenzie S, Wu W-C (2015). Effects of exercise training on cardiorespiratory fitness and biomarkers of cardiometabolic health: a systematic review and meta-analysis of randomized controlled trials. J Am Heart Assoc.

[8] Steeves JA, Murphy RA, Crainiceanu CM, Zipunnikov V, Van Domelen DR, Harris TB (2015). Daily patterns of physical activity by type 2 diabetes definition: comparing diabetes, prediabetes, and participants with normal glucose levels in NHANES 2003–2006. Prev Med Rep.

[9] Hobbs N, Godfrey A, Lara J, Errington L, Meyer TD, Rochester L (2013). Are behavioral interventions effective in increasing physical activity at 12 to 36 months in adults aged 55 to 70 years? A systematic review and meta-analysis. BMC Med.

[10] Mosalman Haghighi Marjan, Mavros Yorgi, Fiatarone Singh Maria A. (2018). The Effects of Structured Exercise or Lifestyle Behavior Interventions on Long-Term Physical Activity Level and Health Outcomes in Individuals With Type 2 Diabetes: A Systematic Review, Meta-Analysis, and Meta-Regression. Journal of Physical Activity and Health.

[11] Wing RR, Bahnson JL, Bray GA, Clark JM, Coday M, Egan C, et al. Long-term effects of a lifestyle intervention on weight and cardiovascular risk factors in individuals with type 2 diabetes mellitus: four-year results of the look AHEAD trial. Arch Intern Med. 2010;170:1566-75. 10.1001/archinternmed.2010.334 PMC3084497 20876408

[12] Umpierre Daniel (2011). Physical Activity Advice Only or Structured Exercise Training and Association With HbA1cLevels in Type 2 Diabetes. JAMA.

[13] Department for Transport (2016). Road use statistics: Great Britain 2016.

[14] Vojnovic I (2006). Building communities to promote physical activity: a multi-scale geographical analysis. Geografiska Annaler Series B Hum Geogr.

[15] de Hartog JJ, Boogaard H, Nijland H, Hoek G (2010). Do the health benefits of cycling outweigh the risks?. Environ Health Perspect.

[16] Maibach E, Steg L, Anable J (2009). Promoting physical activity and reducing climate change: opportunities to replace short car trips with active transportation. Prev Med.

[17] Sahlqvist S, Goodman A, Cooper AR, Ogilvie D (2013). Change in active travel and changes in recreational and total physical activity in adults: longitudinal findings from the iConnect study. Int J Behav Nutr Phys Act.

[18] Laverty AA, Mindell JS, Webb EA, Millett C (2013). Active travel to work and cardiovascular risk factors in the United Kingdom. Am J Prev Med.

[19] Flint E, Cummins S (2016). Active commuting and obesity in mid-life: cross-sectional, observational evidence from UK Biobank. Lancet Diabetes Endocrinol.

[20] Falconer Catherine L, Cooper Ashley R, Flint Ellen (2017). Patterns and correlates of active commuting in adults with type 2 diabetes: cross-sectional evidence from UK Biobank. BMJ Open.

[21] Celis-Morales CA, Lyall DM, Welsh P, Anderson J, Steell L, Guo Y, et al. Association between active commuting and incident cardiovascular disease, cancer, and mortality: prospective cohort study. BMJ. 2017;357. 10.1136/bmj.j1456. 10.1136/bmj.j1456 28424154

[22] Oja P, Mänttäri A, Heinonen A, Kukkonen-Harjula K, Laukkanen R, Pasanen M (1991). Physiological effects of walking and cycling to work. Scand J Med Sci Sports.

[23] De Geus B, Hendriksen I (2015). Cycling for transport, physical activity and health: what about Pedelecs?.

[24] Fishman E, Cherry C (2016). E-bikes in the mainstream: reviewing a decade of research. Transp Rev.

[25] Peterman JE, Morris KL, Kram R, Byrnes WC (2016). Pedelecs as a physically active transportation mode. Eur J Appl Physiol.

[26] Sundfør HB, Fyhri A (2017). A push for public health: the effect of e-bikes on physical activity levels. BMC Public Health.

[27] Cairns S, Behrendt F, Raffo D, Beaumont C, Kiefer C (2017). Electrically-assisted bikes: potential impacts on travel behaviour. Transp Res Part A Policy Pract.

[28] Bourne JE, Sauchelli S, Perry R, Page A, Leary S, England C, et al. Health benefits of electrically-assisted cycling: a systematic review. Int J Behav Nutr Phys Act. 2018;15(116). 10.1186/s12966-018-0751-8. 10.1186/s12966-018-0751-8 PMC6249962 30463581

[29] Cooper A. R., Tibbitts B., England C., Procter D., Searle A., Sebire S. J., Ranger E., Page A. S. (2018). Potential of electric bicycles to improve the health of people with Type 2 diabetes: a feasibility study. Diabetic Medicine.

[30] CSEP. Get Active Questionnaire. Ottawa: Canadian Society for Exercise Physiology; 2017. Available from http://www.csep.ca/home.

[31] Lancaster GA, Dodd S, Williamson PR (2004). Design and analysis of pilot studies: recommendations for good practice. J Eval Clin Pract.

[32] Sim J, Lewis M (2012). The size of a pilot study for a clinical trial should be calculated in relation to considerations of precision and efficiency. J Clin Epidemiol.

[33] Andrews R, Cooper AR, Montgomery AA, Norcross AJ, Peters TJ, Sharp DJ (2011). Diet or diet plus physical activity versus usual care in patients with newly diagnosed type 2 diabetes: the early ACTID randomised controlled trial. Lancet..

[34] Cane J, O’Connor D, Michie S. Validation of the theoretical domains framework for use in behaviour change and implementation research. Implement Sci. 2012;7(37). 10.1186/1748-5908-7-37. 10.1186/1748-5908-7-37 PMC3483008 22530986

[35] Michie S, Atkins L, West R (2014). The Behaviour Change Wheel: a guide to designing interventions.

[36] Avery L., Flynn D., van Wersch A., Sniehotta F. F., Trenell M. I. (2012). Changing Physical Activity Behavior in Type 2 Diabetes: A systematic review and meta-analysis of behavioral interventions. Diabetes Care.

[37] Cradock KA, ÓLaighin G, Finucane FM, Gainforth HL, Quinlan LR, Ginis KA (2017). Behaviour change techniques targeting both diet and physical activity in type 2 diabetes: a systematic review and meta-analysis. Int J Behav Nutr Phys Act.

[38] World Health Organization (2011). Waist circumference and waist-hip ratio: report of a WHO expert consultation.

[39] Matsuda M, DeFronzo RA (1999). Insulin sensitivity indices obtained from oral glucose tolerance testing: comparison with the euglycemic insulin clamp. Diabetes Care.

[40] Ware JE, Sherbourne CD (1992). The MOS 36-item short-form health survey (SF-36): I. conceptual framework and item selection. Med Care.

[41] Schaun GZ (2017). The maximal oxygen uptake verification phase: a light at the end of the tunnel?. Sports Med Open.

[42] Scharhag-Rosenberger F, Carlsohn A, Cassel M, Mayer F, Scharhag J (2011). How to test maximal oxygen uptake: a study on timing and testing procedure of a supramaximal verification test. Appl Physiol Nutr Metab.

[43] Doube M, Kłosowski MM, Arganda-Carreras I, Cordelières FP, Dougherty RP, Jackson JS (2010). BoneJ: free and extensible bone image analysis in ImageJ. Bone..

[44] Rueden CT, Schindelin J, Hiner MC, DeZonia BE, Walter AE, Arena ET (2017). ImageJ2: ImageJ for the next generation of scientific image data. BMC Bioinformatics.

[45] Freedson PS, Melanson E, Sirard J (1998). Calibration of the computer science and applications, Inc. accelerometer. Med Sci Sports Exerc.

[46] Ekelund U, Sepp H, Brage S, Becker W, Jakes R, Hennings M (2006). Criterion-related validity of the last 7-day, short form of the International Physical Activity Questionnaire in Swedish adults. Public Health Nutr.

[47] TROIANO RICHARD P., BERRIGAN DAVID, DODD KEVIN W., MÂSSE LOUISE C., TILERT TIMOTHY, MCDOWELL MARGARET (2008). Physical Activity in the United States Measured by Accelerometer. Medicine & Science in Sports & Exercise.

[48] Procter DS, Page AS, Cooper AR, Nightingale CM, Ram B, Rudnicka AR (2018). An open-source tool to identify active travel from hip-worn accelerometer, GPS and GIS data. Int J Behav Nutr Phys Act.

[49] Neves Andre, Brand Christian (2019). Assessing the potential for carbon emissions savings from replacing short car trips with walking and cycling using a mixed GPS-travel diary approach. Transportation Research Part A: Policy and Practice.

[50] DEFRA (2017). Greenhouse gas reporting - conversion factors 2017.

[51] Sparkes A, Smith B (2014). Qualitative research methods in sport, exercise and health.

[52] Tudor-Locke C, Bell RC, Myers AM, Harris SB, Ecclestone NA, Lauzon N (2004). Controlled outcome evaluation of the First Step Program: a daily physical activity intervention for individuals with type II diabetes. Int J Obes.

