# Supplementary material for: Electrically assisted cycling for individuals with type 2 diabetes mellitus: protocol for a pilot randomized controlled trial
Source: Pilot Feasibility Stud. 2019 Nov 23;5:136. doi: 10.1186/s40814-019-0508-4 (PMC6875029; doi:10.1186/s40814-019-0508-4)
Supplement: Supplementary file 2 — Additional file 2. Behavioural Intervention. [file 40814_2019_508_MOESM2_ESM.docx]

| **Supplementary File 1.** Design of the PEDAL-2 intervention | | |
| --- | --- | --- |
| **Session** | **Intervention content** | **Associated behaviour change technique** |
| **E-bike training phase** | | |
| Session 1 | One-to-one physical training on e-bike with Life Cycle UK instructor including using gears, how to ride in traffic etc. Training will follow National Skills Level 1 and 2 | 4.1 Instruction on how to perform behaviour  6.1 Demonstration of behaviour  8.1 Behavioural practice/rehearsal |
|  | Instructor to provide participant with feedback on their e-cycling | 2.2 Feedback on the behaviour |
|  | Instructor to provide positive encouragement to participant throughout the session | 3.1 Social support (unspecified) |
|  | Participant provided with log book (online or paper) and encouraged to record their e-cycling activity | 2.3 Self-monitoring of behaviour |
|  | Participant asked to set specific e-cycling goals for the upcoming e-bike loan period | 1.1 Goal-setting (behaviour) |
|  | Instructor to provide participant with information on potential health and emotional benefits associated with physical activity and specifically e-cycling. The potential environmental consequences of e-cycling will also be discussed. | 5.1 Information about health consequences  5.4 Information about emotional consequences  5.3 Information about social and environmental consequences |
|  | Instructor to show participants video of two individuals with type 2 diabetes mellitus who have been converted to regular e-bike users | 6.1 Demonstration of behaviour  16.3 Vicarious consequences |
|  | Instructor to encourage participant to think about when and where they plan to ride the e-bike and make ride plans | 1.4 Action planning |
| Session 2  *Optional | One-to-one physical training on e-bike with Life Cycle instructor following National Skills Level 3 | 4.1 Instruction on how to perform behaviour  6.1 Demonstration of behaviour  8.1 Behavioural practice/rehearsal |
|  | Instructor to provide participant with feedback on their e-cycling | 2.2 Feedback on behaviour |
|  | Participant encouraged to record their e-cycling in the log book | 2.3 Self-monitoring of behaviour |
|  | Participant encouraged to identify potential barriers to e-cycling and come up with ways to overcome these barriers | 1.2 Problem solving |
|  | Participant provided with helmet, panniers and lights for the duration of the e-bike loan period and cycling maps | 12.5 Adding objects to the environment |
|  | Participant connected to other participants via social media (WhatsApp Group) and provided with information on Life Cycle UK social rides.  Participants also advised to connect with friends and family and to inform them of their goals to build support | 3.1 Social support (unspecified) |
|  | Participant provided with details of bike breakdown service which can be utilized throughout the intervention period | 3.2 Social support (practical) |
| **E-bike loan phase** | | |
| Session 3  Location of participants choice | Participant and instructor ride together. Participant to decide on where they would like to practice riding – could involve trying a new route or trying a busy road | 4.1 Instruction on how to perform behaviour  6.1 Demonstration of behaviour  8.1 Behavioural practice/rehearsal |
|  | Instructor to provide feedback to the participant on their riding | 2.2 Feedback on behaviour |
|  | Instructor and participant to review past 4-weeks e-cycling behaviour using log book. Instructor to provide positive encouragement and to encourage participant to focus on past success | 3.1 Social support (unspecified)  15.3 Focus on past success |
|  | Review goals and encourage participant to amend if necessary | 1.5 Review behaviour goals |
|  | Review barriers to e-cycling that have arisen and how these were overcome/could be overcome in the future | 1.2 Problem solving |
|  | Participant encouraged to plan where and when they want to ride in the future. | 1.4 Action planning |
| Session 4  Telephone | Review of e-cycling behaviour over the past month and instructor to provide feedback | 2.2 Feedback on behaviour |
|  | Review goals and encourage participant to amend if necessary | 1.5 Review behaviour goals |
|  | Instructor to focus on successes and provide positive encouragement | 3.1 Social support (unspecified)  15.3 Focus on past success |
|  | Review of barriers that have arisen and discussion on how these were overcome/plan ways to overcome these | 1.2 Problem solving |
|  | Discuss how and where participants plan to ride in the final 4 weeks | 1.4 Action planning |
|  | Recap on the potential health and emotional benefits participant may experience through e-cycling | 5.1 Information about health consequences  5.4 Information about emotional consequences |
|  | Participant reminded of the importance of self-monitoring e-cycling behaviour and encouraged to continue recording behaviour | 2.3 Self-monitoring of behaviour |
